# Gender differences in the relationship between sleep and childhood traumas

**DOI:** 10.1192/j.eurpsy.2023.611

**Published:** 2023-07-19

**Authors:** R. Fusco, S. Kulkarni

**Affiliations:** ^1^Social Work, University of Georgia, Athens; ^2^Social Work, UNC Charlotte, Charlotte, United States

## Abstract

**Introduction:**

Sleep is one of the most important factors for well-being. Numerous studies have linked poor sleep to negative consequences such as depression (Guo et al., 2014), substance use (Comasco et al., 2010), obesity (Wu et al., 2015), and suicidality (Liu & Buysse, 2006). Understanding the causes of poor sleep is an important public health concern. Childhood trauma can have a lifelong influence on mental health and development. However, many examinations of childhood trauma focus on the number of experiences and not the impact of specific experiences. Research has shown gender differences in both insomnia and childhood maltreatment outcomes (Lee et al., 2014); however, less attention has been paid to the potential role of gender in the link between other types of childhood trauma and sleep disturbance.

**Objectives:**

The goal of the current study is to understand the role of gender in the relationship between childhood traumas and sleep problems among a community sample of emerging adults. This research aims to build on and extend previous work in three different ways: 1) add to the literature on childhood trauma among emerging adults; 2) examine specific types of childhood traumas; and 3) expand the categories to explore the role of some less studied adversities.

**Methods:**

The study included a sample of 211 young adults from an urban community. Traumas were measured with the Brief Trauma Questionnaire (BTQ; Schnurr et al., 1999) and sleep was measured with the Pittsburgh Sleep Quality Index (Buysse et al., 1989). Logistic regression was used to calculate odds ratios for the relationship between sleep problems and five traumas (involved in a serious accident, experienced serious injury, violent death of a close family member or friend, witnessed someone’s serious injury or death, and natural disaster).

**Results:**

Results showed that males and females experienced similar rates of trauma. Trauma experienced during childhood was associated with an increased prevalence of poor sleep. In the male model, being involved in a serious accident (OR=1.47), experiencing serious injury (OR=1.24), and experiencing a natural disaster (OR=1.11) were all significantly associated with poor sleep. In the female model, the violent death of a close family member or friend (OR=2.02), witnessing someone’s serious injury or death (OR=1.78), and experiencing a natural disaster (OR=1.69) were all significantly associated with poor sleep.

**Conclusions:**

Traumatic events may impact men and women differently. Women in the study showed greater sleep problems in the wake of childhood traumatic events. They also responded more strongly to events that they saw happening to other people, while men were more greatly affected by things that happened directly to them. Natural disasters are a relatively common event that has a strong impact on sleep. Intervention efforts addressing trauma and poor sleep should be aware of gender differences for greater efficacy.

**Disclosure of Interest:**

None Declared

